# “磨玻璃影”特征的早期肺腺癌中MPP成分的突变特征分析及ZNF469基因的探索

**DOI:** 10.3779/j.issn.1009-3419.2023.106.23

**Published:** 2023-12-20

**Authors:** Youtao XU, Qinhong SUN, Siwei WANG, Hongyu ZHU, Guozhang DONG, Fanchen MENG, Zhijun XIA, Jing YOU, Xiangru KONG, Jintao WU, Peng CHEN, Fangwei YUAN, Xinyu YU, Jinfu JI, Zhitong Li, Pengcheng Zhu, Yuxiang Sun, Tongyan LIU, Rong YIN, Lin XU

**Affiliations:** 210009 南京，南京医科大学附属肿瘤医院，江苏省肿瘤医院胸外科，江苏省肿瘤防治研究所，江苏省恶性肿瘤分子生物学及转化医学重点实验室; Department of Thoracic Surgery, the Affiliated Cancer Hospital of Nanjing Medical University, Jiangsu Cancer Hospital, Jiangsu Institute of Cancer Research, Jiangsu Key Laboratory of Molecular and Translation Cancer Research, Nanjing 210009, China

**Keywords:** 肺肿瘤, 磨玻璃影, 微乳头成分, 突变特征分析, ZNF469基因, Lung neoplasms, Ground-glass opacities, Micropapillary, Mutational signatures analysis, ZNF469 gene

## Abstract

**背景与目的** 目前，肺癌依然是我国发病率和死亡率最高的恶性肿瘤。而在早期肺腺癌（lung adenocarcinoma, LUAD）中，微乳头（micropapillary, MPP）成分尤其常见，且通常表现出高侵袭性，其与早期转移、淋巴浸润的风险以及患者的5年生存率显著相关。本研究旨在探究以磨玻璃影（ground-glass opacities, GGOs）为特征的早期LUAD中MPP成分和非MPP成分的异同，寻找MPP成分所特有的突变特征，并分析锌指蛋白家族的ZNF469基因与早期LUAD预后以及免疫浸润的关系。**方法** 收集31例LUAD恶性肺结节，采用显微解剖法将其分为成对的MPP和非MPP成分。对早期恶性肺结节组分进行全外显子组测序（whole-exome sequencing, WES），利用maftools、非负矩阵分解（Nonnegative Matrix Factorization, NMF）法、Sigminer等方法进行突变特征分析，以揭示侵袭性LUAD中MPP组分相比于其他肿瘤组织所特有的基因组突变特征。利用癌症基因组图谱（The Cancer Genome Atlas, TCGA）的LUAD数据库中ZNF469的表达情况，探讨其与肺癌预后的关系；利用GeneMANIA数据库以及基因本体（Gene Ontology, GO）、京都基因与基因组百科全书（Kyoto Encyclopedia of Genes and Genomes, KEGG）富集分析探索LUAD中与ZNF469相关基因的互作网络及信号通路；利用TIMER和TISIDB数据库分析ZNF469表达与LUAD中免疫细胞浸润水平的相关性。**结果** MPP成分具有较多的基因组变异，相比于非MPP成分的肿瘤组织，癌症体细胞突变目录（Catalogue of Somatic Mutations in Cancer, COSMIC）的13号突变特征（胞苷脱氨酶家族，APOBEC）是MPP成分所特有的，这提示其参与了MPP成分对LUAD早期侵袭过程的促进作用；并且APOBEC特征高的MPP样本具有更高的肿瘤突变负荷（tumor mutational burden, TMB），提示这些患者更能从免疫治疗中获益。LUAD中突变ZNF469的表达高于正常组织，与LUAD患者的不良预后有关。基因互作网络分析以及GO和KEGG富集分析发现，COL6A1、COL1A1、COL1A2、TGFB2、MMP2、COL8A2、C2CD4C等与ZNF469具有相互作用，且主要与编码胶原蛋白、参与细胞外基质构成有关。ZNF469表达与肿瘤的免疫浸润呈正相关。**结论** 本研究揭示了中国人群侵袭性LUAD中MPP成分的特有突变特征，并发现突变ZNF469的高表达影响LUAD预后与免疫浸润，推测ZNF469可作为LUAD潜在的诊断及预后生物标志物。

## Abstract (en)

**Background and objective** In China, lung cancer remains the cancer with the highest incidence and mortality rate. Among early-stage lung adenocarcinomas (LUAD), the micropapillary (MPP) component is prevalent and typically exhibits high aggressiveness, significantly correlating with early metastasis, lymphatic infiltration, and reduced five-year survival rates. Therefore, the study is to explore the similarities and differences between MPP and non-micropapillary (non-MPP) components in malignant pulmonary nodules characterized by GGOs in early-stage LUAD, identify unique mutational features of the MPP component and analyze the relationship between the ZNF469 gene, a member of the zinc-finger protein family, and the prognosis of early-stage LUAD, as well as its correlation with immune infiltration. **Methods** A total of 31 malignant pulmonary nodules of LUAD were collected and dissected into paired MPP and non-MPP components using microdissection. Whole-exome sequencing (WES) was performed on the components of early-stage malignant pulmonary nodules. Mutational signatures analysis was conducted using R packages such as maftools, Nonnegative Matrix Factorization (NMF), and Sigminer to unveil the genomic mutational characteristics unique to MPP components in invasive LUAD compared to other tumor tissues. Furthermore, we explored the expression of the ZNF469 gene in LUAD using The Cancer Genome Atlas (TCGA) database to investigate its potential association with the prognosis. We also investigated gene interaction networks and signaling pathways related toZNF469 in LUAD using the GeneMANIA database and conducted Gene Ontology (GO) and Kyoto Encyclopedia of Genes and Genomes (KEGG) enrichment analysis. Lastly, we analyzed the correlation between ZNF469 gene expression and levels of immune cell infiltration in LUAD using the TIMER and TISIDB databases. **Results** MPP components exhibited a higher number of genomic variations, particularly the 13^th^ COSMIC (Catalogue of Somatic Mutations in Cancer) mutational signature characterized by the activity of the cytidine deaminase APOBEC family, which was unique to MPP components compared to non-MPP components in tumor tissues. This suggests the potential involvement of APOBEC in the progression of MPP components in early-stage LUAD. Additionally, MPP samples with high similarity to APOBEC signature displayed a higher tumor mutational burden (TMB), indicating that these patients may be more likely to benefit from immunotherapy. The expression ofZNF469 was significantly upregulated in LUAD compared to normal tissue, and was associated with poor prognosis in LUAD patients (P<0.05). Gene interaction network analysis and GO/KEGG enrichment analysis revealed that COL6A1, COL1A1, COL1A2, TGFB2, MMP2, COL8A2 and C2CD4C interacted with ZNF469 and were mainly involved in encoding collagen proteins and participating in the constitution of extracellular matrix. ZNF469 expression was positively correlated with immune cell infiltration in LUAD (P<0.05). **Conclusion** The study has unveiled distinctive mutational signatures in the MPP components of early-stage invasive LUAD in the Asian population. Furthermore, we have identified that the elevated expression of mutated ZNF469 impacts the prognosis and immune infiltration in LUAD, suggesting its potential as a diagnostic and prognostic biomarker in LUAD.

2001年文献统计数据^[1]^显示，在中国的恶性肿瘤中，肺癌依然是发病率和死亡率最高的一种癌症，而肺腺癌（lung adenocarcinoma, LUAD）约占所有肺癌病例的60%。随着肺癌筛查的逐步推广与普及，早期诊断的LUAD患者数量逐渐增多。据报道^[2]^，有相当一部分早期LUAD患者在接受临床干预后的5年内仍然会出现复发或转移情况。在这一阶段，肺癌组织中不同的细胞成分的组合，如鳞状细胞、黏液状细胞、腺泡状细胞、乳头状细胞、实性细胞和微乳头（micropapillary, MPP）状细胞，已被证明是预后评估的重要因素^[3]^。其中，MPP状成分通常表现出更具侵袭性的分子标志，其在肿瘤细胞中的比例与早期转移、淋巴浸润的风险以及患者的5年生存率显著相关^[4⇓⇓-7]^。在肺部恶性结节中，MPP成分尤其常见，这些结节通常呈现出磨玻璃影的特征，而MPP成分主要分布在肿瘤边缘，表现出意想不到的由内向外的生长模式^[8]^。这些细胞在与周围组织的交界处具有根尖分泌特性，其分泌的物质包括促进肿瘤增殖和转移的因子。这些细胞特性在很大程度上决定了肿瘤的行为和恶性程度。而MPP成分在某些特定的肿瘤中伴随着特征性突变，例如，表皮生长因子受体（epidermal growth factor receptor, EGFR）和V-raf鼠肉瘤病毒癌基因同源体B（vrafmurine sarcoma viral oncegene homolog B, BRAF）基因在MPP样本中的突变频率较高^[9]^。目前已有的面板测序研究^[10]^也指出，与其他成分相比，MPP成分具有更强的基因组不稳定性和更高的遗传异质性，并且携带着特定的突变基因，这可能与LUAD不同发展阶段中亚克隆突变的积累有关。尽管上述研究结果非常有价值，但在早期LUAD中，关于MPP成分在基因组水平上的细胞突变的详细信息仍然有限。因此，本研究进行了亚洲人群中MPP和非MPP亚型的基因组学的研究，旨在揭示MPP亚型肿瘤在生长过程中的特殊突变特征，并试图找到与患者的临床预后相关的新的潜在基因，以对早期微乳头型LUAD的治疗提供指导作用。

众所周知，癌症主要是由基因组DNA的体细胞突变引起的。根据基因组改变的大小和特征，这些癌症相关的DNA改变可分为以下四种类型：单碱基替换（single base substitution, SBS）、小插入和缺失（insertion and deletion, INDEL）、包括易位/倒位在内的结构改变，以及拷贝数改变（copy number alteration, CNA）。基因组DNA突变特征是一种重复出现的基因组模式，是癌细胞一生中积累的诱变过程的印记。基因组突变特征分析不仅可以提供突变过程信息，还可以为癌症精准医疗提供生物标志物。Cosmic SBS特征分析已被广泛研究，并代表了其他类型的特征研究的原型，并且突变特征的分析正在成为癌症基因组学的常规手段，对癌症发病机制、分类和预后均有影响^[11]^。因此，本研究也会着重对两种成分进行突变特征分析。

此外，有报道^[12]^称MPP亚型与免疫疗法疗效之间存在相关性，因此我们也试图寻找新的免疫浸润相关基因，并评估其在LUAD预后中的作用。我们通过样本的突变景观筛选得出了ZNF469这一基因，目前已知的文献研究^[13]^表明其隶属于C2H2-TYPE锌指蛋白家族，参与编码锌指蛋白，可能作为一种转录因子调节参与胶原蛋白合成。目前关于ZNF469的研究大多集中在脆性角膜综合征^[14]^，而关于癌症的研究屈指可数，仅有少数文献报道过其在胃癌中存在突变^[15]^，作为COL1A2的相似基因在食管癌免疫浸润方面可能有一定影响^[16]^，这为我们研究其在LUAD中的作用提供了思路。本研究也旨在验证其对于LUAD的预后影响以及与免疫浸润的相关性，为其之后在指导预后及免疫治疗方面的作用奠定基础。

## 1 资料与方法

### 1.1 样本与显微切割

本项研究选取了2016年2月至2021年2月在南京医科大学附属肿瘤医院进行肺癌手术治疗的31例LUAD病例的肺部恶性肿瘤组织样本进行分析，目的是探寻这些肿瘤组织中的突变特性。通过手术得到的LUAD样本，均含有MPP成分，并被病理学诊断确认。根据美国癌症联合委员会（American Joint Committee on Cancer, AJCC）第八版的分期标准，这些样本均定为I期。样本中的MPP成分由两位经验丰富的病理学家在显微镜下观察识别，并进行显微切割，从而从每份样本中分离出含有和不含有MPP的部分。同时，我们还收集了这31例患者的外周血单核细胞（peripheral blood mononuclear cell, PBMC）作为基因变异的参照。研究流程均获得了南京医科大学伦理委员会的批准，研究参与者也已充分知情并同意。相关的临床信息被整理在表1中。

**表1:** 31例LUAD病例的临床信息

Patient ID	Age (yr)	Gender	TNM	Stage	MPP component content
1	78	Male	T1bN0M0	IA	15%
2	55	Male	T2aN0M0	IB	10%
3	58	Female	T2aN0M0	IB	20%
4	70	Male	T1aN0M0	IA	10%
5	65	Male	T1aN0M0	IA	20%
6	76	Female	T2aN0M0	IB	20%
7	58	Female	T1cN0M0	IA	20%
8	56	Female	T1cN0M0	IA	5%
9	59	Male	T2aN0M0	IB	10%
10	57	Female	T2aN0M0	IB	5%
11	64	Female	T2aN0M0	IB	30%
12	38	Male	T2aN0M0	IB	20%
13	59	Female	T1bN0M0	IA	40%
14	55	Female	T1aN0M0	IA	20%
15	72	Female	T1aN0M0	IA	30%
16	58	Male	T2aN0M0	IB	20%
17	54	Female	T1aN0M0	IA	30%
18	65	Male	T1aN0M0	IA	60%
19	59	Male	T1bN0M0	IA	20%
20	63	Female	T1bN0M0	IA	30%
21	66	Male	T1bN0M0	IA	60%
22	50	Female	T1bN0M0	IA	40%
23	63	Female	T1bN0M0	IA	30%
24	64	Female	T1cN0M0	IA	30%
25	60	Male	T1cN0M0	IA	40%
26	55	Male	T2aN0M0	IB	50%
27	55	Female	T2aN0M0	IB	30%
28	56	Male	T2aN0M0	IB	40%
29	67	Male	T2aN0M0	IB	30%
30	64	Female	T2aN0M0	IB	60%
31	69	Female	T2aN0M0	IB	70%

LUAD: lung adenocarcinoma; TNM: tumor-node-metastasis; MPP: micropapillary.

### 1.2 全外显子组测序（whole-exome sequencing, WES）

使用FastPure FFPE DNA分离试剂盒和FastPure血液DNA分离试剂盒从组织和血液样本中提取DNA。使用Qubit®3.0荧光计（Q33216, Life Technologies, Carlsbad, CA, USA）测量DNA样本浓度。按照Agilent SureSelectXT目标富集系统的方法和步骤制备DNA小片段文库。文库构建完成后，使用Qubit 3.0进行初始量化，使用Agilent 2100测量库的插入片段大小。插入片段尺寸达到预期后，采用Bio-RAD CFX 96荧光定量聚合酶链式反应（polymerase chain reaction, PCR）仪和Bio-RAD KIT iQ SYBR GRN进行Q-PCR，以准确定量文库有效浓度（有效文库浓度>10 nmol/L），保证文库质量。文库通过质控后，在Illumina HiSeq测序平台上按照厂家的说明书运行双端测序程序（PE150）生成FASTQ文件格式的原始数据。本项目测序数据质量采用FastQC v0.11.7软件进行评估，并使用BWA等软件与参考基因组进行比较。使用GATK软件进行变异检测，使用ANNOVAR软件进行单核苷酸多态性（single nucleotide polymorphism, SNP）和InDel标注^[17⇓⇓-20]^。

### 1.3 突变特征分析（mutational signatures analysis）

基于WES数据所得到的maf文件，使用非负矩阵分解（Nonnegative Matrix Factorization, NMF）包对MPP样本、非MPP样本以及tracerx样本分别进行非负矩阵的分解^[21]^，并利用sigminer包绘制出每种样本最具特征性的signatures并与COSMIC signatures进行比对^[11]^。使用GenomicRanges包将maf文件转换为SomaticSignatures包所需要的文件格式，然后分别计算每一个样本的突变特征与60个COSMIC signatures的余弦相似度（cosine similarity）^[22,23]^，筛选出MPP样本和非MPP样本中有统计学差异（P<0.05）的signatures来验证NMF得出的结果；并且结果在TRACER X 100样本队列中得到验证^[24]^。

### 1.4 免疫细胞浸润的相关性分析及基因互作网络分析

本研究基于TIMER 2.0数据库来评估ZNF469表达与免疫细胞浸润的关系^[25]^，并采用Spearman法研究ZNF469与免疫细胞浸润的相关性，包括肿瘤相关成纤维细胞、CD4^+^ T细胞、内皮细胞、巨噬细胞、单核细胞和中性粒细胞。基于TISIDB数据库分析ZNF469丰度与免疫亚型的关系以及与免疫细胞浸润的相关性^[26]^。

### 1.5 统计分析

使用R（v4.2.2）进行统计分析。对于组间连续变量的比较，采用Wilcoxon秩和检验。Survminer、ggplot2包用于可视化分析，GOplot包用于基因本体（Gene Ontology, GO）和京都基因与基因组百科全书（Kyoto Encyclopedia of Genes and Genomes, KEGG）富集分析的可视化弦图。P<0.05为差异具有统计学意义。

## 2 结果

### 2.1 恶性肺结节中MPP和非MPP成分突变景观及突变特征分析

基于已有的WES数据，描绘了突变的全景，在LUAD患者中，错义突变是主要的突变分型（Variant Classification，图1A、1D），而SNP是主要的突变形式（Variant Type，图1B）。C>T碱基替换是LUAD患者最常见的SNP类型，而C>A次之（图1C）。通过联合对比柱状图（图1E）可以看出，MPP与非MPP成分的基因突变率的差异是明显存在的，MPP组织中的突变要多于非MPP组织。

**图1:**
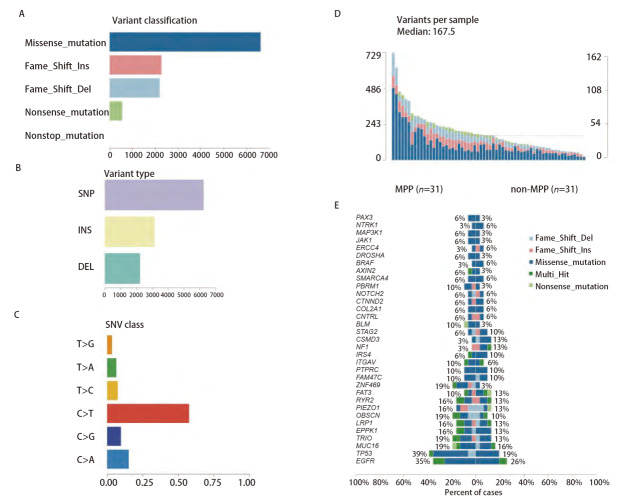
早期LUAD中MPP与非MPP成分的突变景观展示。A-D：错义突变是主要的突变分型，而SNP是主要的突变形式，C>T碱基替换是LUAD患者最常见的SNP类型，而C>A次之；E：MPP与非MPP成分中基因突变率的差异比较。

在癌症的发展过程中，特征性突变模式影响了突变的过程^[11]^，为了研究MPP与非MPP成分在发展过程中的突变模式的差异，使用NMF技术来提取二者的SBS突变特征。突变特征是一种在基因组中观察到的突变的模式或特征，它们可以提供有关细胞DNA损伤和修复过程的信息。每种突变特征都由一组特定类型的DNA突变组成，这些突变可能是由不同的生物学机制引起的，而SBS突变特征主要关注基因组中单个碱基的替代突变^[27]^。对MPP样本和非MPP样本以及TRACER X100中的MPP样本分别进行NMF以进行突变特征的提取。首先按照NMF的说明，将计算得出的cophenetic曲线下降最快的前一个点的值作为样本的特征分解数目，MPP为3，非MPP为3，TRACER X100中的MPP为2（图2A-2C），随后进行了非负矩阵的构建（图2D-2F），可见分解结果的冗杂偏倚较少，效果较好。选取与COSMIC相似度最高的作为每一个的突变特征，根据每个突变特征对于每一例样本的相对贡献度绘制了突变特征图谱（图3A-3D），可以看到MPP与非MPP之间存在差异，其中MPP样本中的第3个突变特征与COSMIC 13号SBS突变特征（胞苷脱氨酶APOBEC家族的活性）最为相似（相似度达0.82）。为了进一步得出MPP成分相较于非MPP成分的独有的突变特征，又用SomaticSignatures包将2种成分中的每一例样本单独与COSMIC数据库的96个SBS突变特征进行了比对，并且分别单独计算了余弦相似度^[23]^（图2G），然后对两种成分样本进行配对检验，显示具有统计学差异（P<0.05）的7个突变特征（图2H），分别是2号、5号、10a号、13号、26号、33号以及46号。其中包含的13号突变特征，与上述结果吻合，由此得出这一突变特征是MPP成分相对于其他肿瘤组织所特有的。

**图2:**
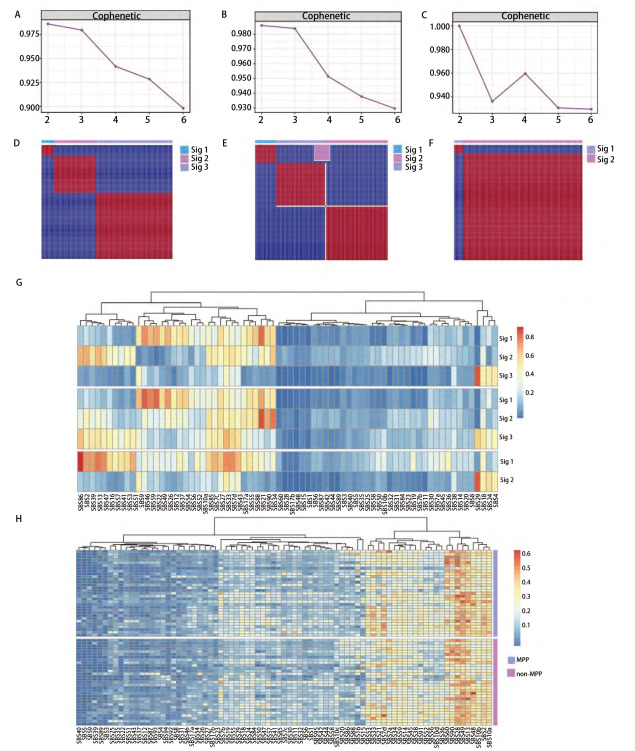
MPP与非MPP成分的突变特征提取过程。A-C：非负矩阵分解中cophenetic曲线的绘制以及rank值的确定；D-F：非负矩阵的构建；G：基于非负矩阵分解得出的三种成分的突变特征情况；H：MPP与非MPP成分配对检验的差异情况。

**图3:**
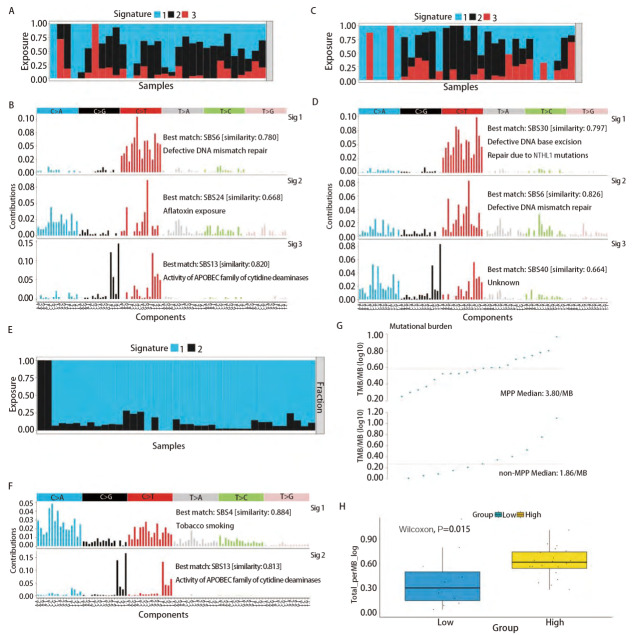
不同成分的突变特征图谱以及不同特征下肿瘤突变负荷的差异。A、C、E：每个突变特征对于每一例样本的相对贡献度；B、D、F：MPP、非MPP以及TRACER X 100中MPP成分的突变特征展示；G，H：以与13号特征的相似度高低为分组，31例MPP成分中肿瘤突变负荷的差异（P=0.015）。

之后在TRACER X 100数据集中对此发现进行验证^[24]^，可以看到2个突变特征（图3E、3F），除去特征1与烟草暴露以外，特征2与胞苷脱氨酶APOBEC家族的活性最相关（相似度达0.813）。由此，验证了APOBEC家族在早期LUAD MPP成分中的独特地位，提示了其对于MPP的发生发展起到了促进作用。

除此以外，又对已有的31个MPP样本按照与COSMIC 13号特征的相似度高低进行了分组，以查看它们的肿瘤突变负荷（tumor mutational burden, TMB）是否具有差异（图3G、3H）。结果显示，相似度高组的TMB高于低组，二者差异具有统计学意义（P=0.015）。

### 2.2 ZNF469在LUAD中高表达且与不良预后相关

基于在MPP和非MPP样本中呈现出的突变率的差异（MPP样本中是19%，非MPP样本是3%），观察了ZNF469在泛癌里配对的肿瘤与正常组织中的表达情况（图4A），对于大多数癌种，其在肿瘤组织中的表达明显高于正常组织（P<0.05）。本研究单独探讨了ZNF469在LUAD中的表达情况以及其表达对LUAD预后的影响，发现LUAD中高表达（图4B，P=8.8e-05），且高表达情况下对LUAD的预后具有明显的不良影响（图4C，P=0.0041）。随后又在泛癌中观察了ZNF469突变与否的表达高低差异（图4D，P=0.0043）以及对泛癌预后的影响（图4E，P=0.0059），结果亦然。结合突变景观分析，ZNF469突变和高表达有作为LUAD不良预后预测指标的潜质。

**图4:**
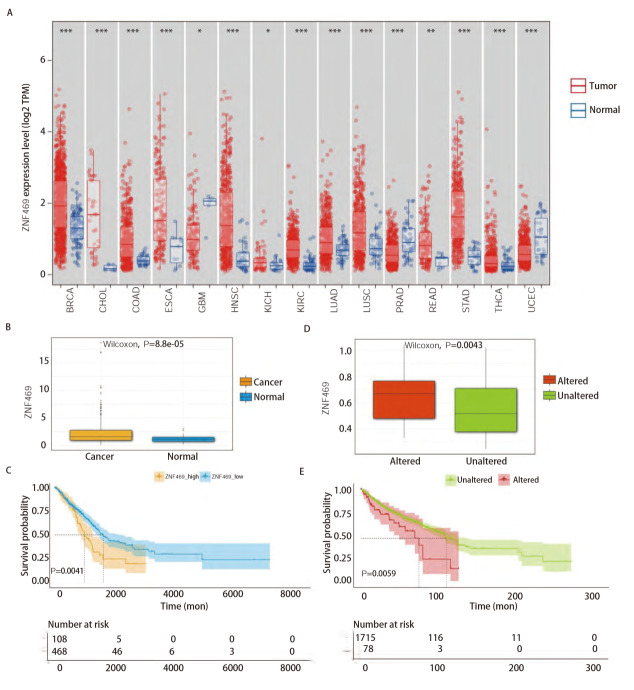
ZNF469在癌症中的表达以及预后情况。A：TCGA pan-cancer数据库肿瘤组织及配对正常组织中ZNF469的表达情况；B：LUAD组织与正常组织中ZNF469表达比较（P=8.8e-05）；C：LUAD中以ZNF469表达水平分组的生存曲线（P=0.0041）；D：泛癌中突变与非突变ZNF469的表达比较（P=0.0043）； E：泛癌中以ZNF469突变与否分组的生存曲线（P=0.0059）。*P<0.05，**P<0.01，***P<0.001。

### 2.3 ZNF469基因富集分析

在LUAD中以ZNF469的表达高低作为分组因素进行差异分析筛选出差异基因，进行了KEGG和GO富集分析（图5A，5B），功能差异较为明显。如图所示ZNF469上调相关基因功能主要集中在细胞外基质（tumor extracellular matrix, ECM）构成、细胞与基质连接、局部黏连以及癌症中的蛋白多糖；而下调相关基因功能则主要集中在补体和凝血级联反应、细胞脂肪酸代谢以及冠状病毒疾病，提示ZNF469与肿瘤ECM存在关联。

**图5:**
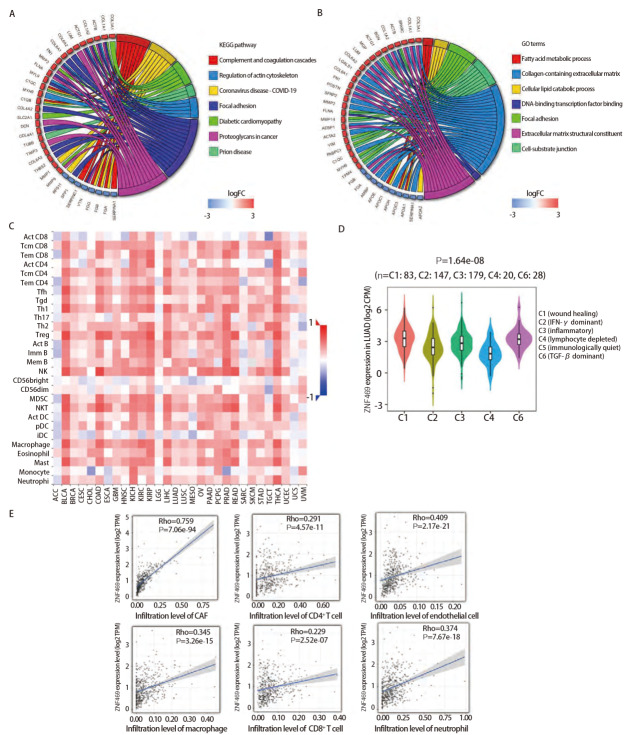
ZNF469的基因富集分析以及其表达与免疫浸润的相关性。A：ZNF469高低分组差异基因KEGG富集分析；B：ZNF469高低分组差异基因GO富集分析；C: 泛癌中不同癌种的ZNF469丰度与浸润淋巴细胞的相关性；D：LUAD中ZNF469表达与免疫亚型的关系；E：LUAD中ZNF469表达和各种免疫细胞浸润水平的相关性散点图。

### 2.4 ZNF469与免疫细胞浸润表达具有相关性

为进一步考察ZNF469对于肿瘤的作用以及影响，分析了ZNF469表达水平与肿瘤免疫细胞浸润的相关性。首先是泛癌中不同癌种下ZNF469丰度与免疫细胞浸润的相关性分布（图5C），可以看到除了个别癌种，如肾上腺皮质癌（adrenocortical carcinoma, ACC）以外 ，ZNF469的表达与大部分常见的肿瘤淋巴细胞浸润情况呈正相关。我们研究了LUAD中ZNF469在不同免疫亚型的分子分型中的表达情况（图5D），发现其在C1（伤口愈合）、C2（IFN-γ占优势）、C3（炎症）、C4（淋巴细胞减少）和C6（TGF-β占优势）亚型中的表达特征存在统计学差异（P=1.64e-08），其中C1亚型表达量最高，这与ZNF469在胶原以及基质形成中发挥的作用相呼应，也验证了此前富集分析的结果。我们还在LUAD中对单个免疫细胞亚型逐个进行分析（图5E），列举了几个常见的重要免疫细胞，可见ZNF469丰度与CD4^+^ T细胞、CD8^+ ^T细胞、巨噬细胞、中性粒细胞、肿瘤相关成纤维细胞（cancer associated fibroblasts, CAF）以及内皮细胞浸润程度均呈正相关且差异具有统计学意义（P<0.05），尤其以CAF相关性为最高（r=0.759），一定程度上验证了ZNF469的肿瘤外基质生成功能。

## 3 讨论

本研究中首先利用突变图谱展示了恶性肺结节中MPP和非MPP的突变景观，展示了突变的分布情况，然后通过NMF提取了两种成分特有的SBS突变特征与COSMIC 96种SBS进行比对，并对两种成分进行配对检验得出了具有显著差异的7个特征；并且在TRACER X 100病例数据库里进行验证，最终确定了13号SBS特征--胞苷脱氨酶APOBEC家族的活性，这是早期LUAD中MPP特有的突变特征^[28,29]^，提示了APOBEC家族基因在早期LUAD MPP的发生发展中起到了作用。此外，对31个MPP样本按照与COSMIC 13号特征的相似度高低进行了分组，发现其TMB具有显著差异。目前已有的一些研究^[30⇓-32]^表明，TMB是非小细胞肺癌免疫检查点抑制剂（immune checkpoint inhibitor, ICI）治疗的预测因子，因此上述结果表明，早期LUAD中MPP分型的患者中，表现出的突变特征与13号特征的相似度越高，其越有可能从ICI治疗中获益。以上发现为日后的MPP成分的LUAD研究和治疗提供了思路。

随后，基于在构建的突变景观中发现的突变率的差异，我们在泛癌中观察了ZNF469的表达图谱，发现其在绝大多数肿瘤中表达量显著高于配对的正常组织，进一步在LUAD中单独研究，除表达差异外，也比较了表达量高低情况下对应的预后，发现表达高者总体生存期明显较短。接着比较了泛癌中ZNF469突变与非突变的表达量情况，发现突变的表达量显著高于非突变的表达量，并且在TCGA泛癌数据库中验证了突变与预后的关系，突变的患者具有更差的预后。这一系列结果均提示了ZNF469突变对预后的不良影响。

我们利用基因互作网络鉴定了ZNF469的共调控基因，包括COL6A1、COL1A1、COL1A2、TGFB2、C2CD4C以及MMP2等，并进行GO和KEGG富集分析，最后总体结果较为一致，显示其基因功能主要集中在编码胶原蛋白、ECM构成和生长因子结合。而ECM在肿瘤发病、进展和转移性扩散过程中又有着明确的作用和影响^[33]^，表明了ZNF469在肿瘤进展过程中所扮演的重要角色。此外，我们研究了ZNF469丰度与肿瘤微环境中免疫细胞浸润的关系，发现在泛癌中二者多数呈正相关，且在LUAD中以B细胞、CD4^+ ^T细胞、CD8^+ ^T细胞、巨噬细胞、中性粒细胞、CAF、内皮细胞以及单核细胞最为突出，体现了ZNF469在肺癌中的免疫学意义。

本次研究通过将肺癌组织进行显微解剖分离出MPP和非MPP组分，然后进行WES，以此对MPP进行突变景观的构建和突变特征的提取，并且从两种成分突变率的差异中我们筛选出了ZNF469这一基因作为研究对象，并且首次通过生物信息学手段分析了其在LUAD中的特异性表达情况、对肺癌预后的不良影响以及功能通路，确定了ZNF469可作为预后指标以及免疫浸润相关标志物的作用。但研究仍存在局限性，例如MPP成分的基因组特征需要在未来更多大量以及多种族患者中得到验证、缺乏对患者的长期随访数据、缺乏非公共的患者转录组数据、未使用细胞实验及动物模型进行验证等。我们将在后续研究中建立细胞和动物模型，通过实验进一步证明该结论。

**Competing interests**

The authors declare that they have no competing interests.

**Author contributions**

Xu YT, Sun QH and Wang SW conceived and designed the study. Zhu HY, Dong GZ, Meng FC, Li ZT, Zhu PC and Sun YX performed the experiments. Xia ZJ, You J and Kong XR analyzed the data. Wu JT, Chen P, Yuan FW, Yu XY and Ji JF contributed analysis tools. Liu TY, Yin R and Xu L provided critical inputs on design, analysis, and interpretation of the study. All the authors had access to the data. All authors read and approved the final manuscript as submitted.
